# Development and implementation of a community-oriented approach to food safety management in a Rural Thai community

**DOI:** 10.1371/journal.pone.0350910

**Published:** 2026-08-27

**Authors:** Vivat Keawdounglek, Anuttara Hongthong, Warapon Paenkhokuard

**Affiliations:** Program of Environmental Health, School of Health Science, Mae Fah Luang University, Chiang Rai, Thailand; Mzuzu University Faculty of Environmental Sciences, MALAWI

## Abstract

Food safety management in rural communities remains challenging because of fragmented governance, informal food systems, and limited stakeholder integration. This study aimed to develop and conduct the early implementation of a Community-Oriented Approach to Food Safety Management (COAFM) in a rural community in northern Thailand. A mixed-method implementation research design was conducted in Wiang Kaen District, Chiang Rai Province, Thailand, integrating community context analysis, food hazard assessment, participatory co-design, and early implementation within an Input–Process–Output framework. Qualitative data were collected through in-depth interviews with key stakeholders, while quantitative data were obtained from a household survey involving 204 households. Chemical and microbiological contamination assessments were conducted in eight food establishments, and binary logistic regression was performed to identify factors associated with household food safety knowledge. The findings showed that the community food system combined household food production with dependence on externally sourced food products, particularly animal-based foods, potentially increasing food safety risks beyond community-level control. Stakeholder awareness of institutional food safety support mechanisms was limited and binary logistic regression identified gender, age, and rice production as significant factors associated with household food safety. Food contamination levels were generally low, although pesticide residues were detected in garlic samples obtained from external markets. The participatory co-design process also resulted in the establishment of a Community Food Safety Committee to strengthen local governance, stakeholder collaboration, and the implementation of food safety activities. Overall, the findings suggest that COAFM provides a feasible and evidence-informed implementation framework that integrates community context assessment, stakeholder engagement, and participatory governance to strengthen food safety management in rural communities. Tailoring interventions according to household characteristics and reinforcing local governance mechanisms may further enhance the sustainability of food safety management in a community.

## Introduction

Food safety remains a major public health concern worldwide, particularly in low- and middle-income countries (LMICs), where food systems are often informal and locally embedded [1–3]. Studies have reported low levels of food safety knowledge among food handlers, for example, in beef shops in southern Ethiopia [4]. In Nigeria, school meal preparation has been associated with a high risk of food contamination due to inadequate hand hygiene practices before handling ready-to-eat foods [5,6]. In addition, chemical contamination, including pesticide residues, and microbial hazards continue to pose significant risks to human health, especially among vulnerable populations such as children and the elderly [7]. For instance, contamination of drinking water with *Escherichia coli* (*E. coli*) has been reported in rural areas, contributing to outbreaks of waterborne diseases [8]. Furthermore, fruits and vegetables may be contaminated with pesticide residues due to increased agricultural demand and inappropriate pesticide use, often associated with limited awareness of their health and environmental impacts [9]. Despite ongoing regulatory efforts, ensuring food safety in real-world community settings remains challenging.

A food safety management system (FSMS) is a systematic approach to controlling hazards and ensuring food safety [10] across the food supply chain, from farm to fork [11]. Such systems typically employ structured frameworks, such as Hazard Analysis and Critical Control Points (HACCP) [12], to prevent contamination, protect public health, and ensure regulatory compliance through documented procedures, staff training, and continuous monitoring [13]. In practice, conventional food safety management is largely based on top-down regulatory approaches, including inspections, standards, and enforcement mechanisms [14,15]. While these strategies have improved food safety in formal sectors, they are often insufficient in rural and community-based contexts, where food production, preparation, and consumption are closely integrated with local practices, culture, and economic conditions. In such settings, food safety risks are not solely technical issues, but are also shaped by social and environmental factors, requiring more context-specific and participatory approaches [16–18]. Recent evidence suggests that Community-Oriented Approaches (COA) [19] to public health can enhance the sustainability and effectiveness of interventions by promoting local ownership, stakeholder engagement, and adaptive learning [20–22]. In the context of food safety, this approach shifts the focus from compliance-driven control to a collaborative system development, where communities actively participate in identifying problems, co-designing solutions, and implementing context-appropriate practices [22–24]. According to several studies [17,23–29], community-oriented approaches have been proposed as complementary strategies to conventional food safety systems, particularly in resource-limited settings. Building upon these established participatory principles, the Community-Oriented Approach to Food Safety Management (COAFM) adapts and integrates community-oriented public health concepts into a structured implementation framework which is specifically designed for food safety management in rural communities. Rather than introducing entirely new participatory principles, COAFM operationalizes stakeholder engagement, collaborative decision-making, adaptive learning, and local governance through a systematic process that integrates household food production, household food demand, food safety risk assessment, and community participation to support sustainable food safety management. The COAFM framework provides a practical implementation pathway for community food safety management, encompassing community context assessment, participatory co-design, implementation, monitoring, and continuous improvement.

In Southeast Asia, food safety remains variable, particularly in terms of multisectoral collaboration. Data from the International Health Regulations (IHR) from the World Health Organization [30] indicate that food safety capacity ranges widely across countries, with reported scores varying from approximately 40% to 100%, reflecting moderate, but uneven performance across the region. This variation highlights persistent gaps in coordination and system integration, particularly in rural and community-based settings, where fragmented governance structures may limit the effectiveness of conventional food safety interventions [31–33]. In Thailand, food safety management has been primarily implemented through regulatory and surveillance systems led by public health authorities [34]. However, challenges remain in translating these policies into effective practices at the community level, particularly in rural areas where food production and consumption are closely integrated with local livelihoods and informal markets [35–38]. These gaps highlight the need for context-specific and community-oriented approaches to strengthen food safety implementation at the local level. Therefore, this study aimed to develop and implement a community-oriented approach to food safety management (COAFM) in a rural Thai community, and to examine how participatory processes can support the development of a sustainable and locally embedded food safety system in the context of a rural community.

## Methodology

### Study design and setting

This study employed a sequential mixed-method implementation research design [39] to develop, implement, and evaluate the Community-Oriented Approach for Food Safety Management (COAFM) in a rural community in northern Thailand. The quantitative phase consisted of a household survey to assess food safety knowledge, household food production capacity, and food demand. The qualitative phase involved in-depth interviews with key stakeholders to explore implementation experiences, contextual factors, barriers, facilitators, and recommendations for improving COAFM. The findings from both phases were integrated to inform the development of an intervention and to evaluate the implementation process comprehensively. The study was conducted in Chiang Rai Province, a region with a high incidence of acute foodborne diseases, as reported by the Department of Disease Control, Thailand [40].

Based on the degree of urbanization framework from the United Nations [41], rural areas are characterized by low population density and dispersed settlements. In this study, Wieng Kaen District can be considered a rural setting, with predominantly agricultural livelihoods and strong reliance on community-based food systems.

**Fig 1:**
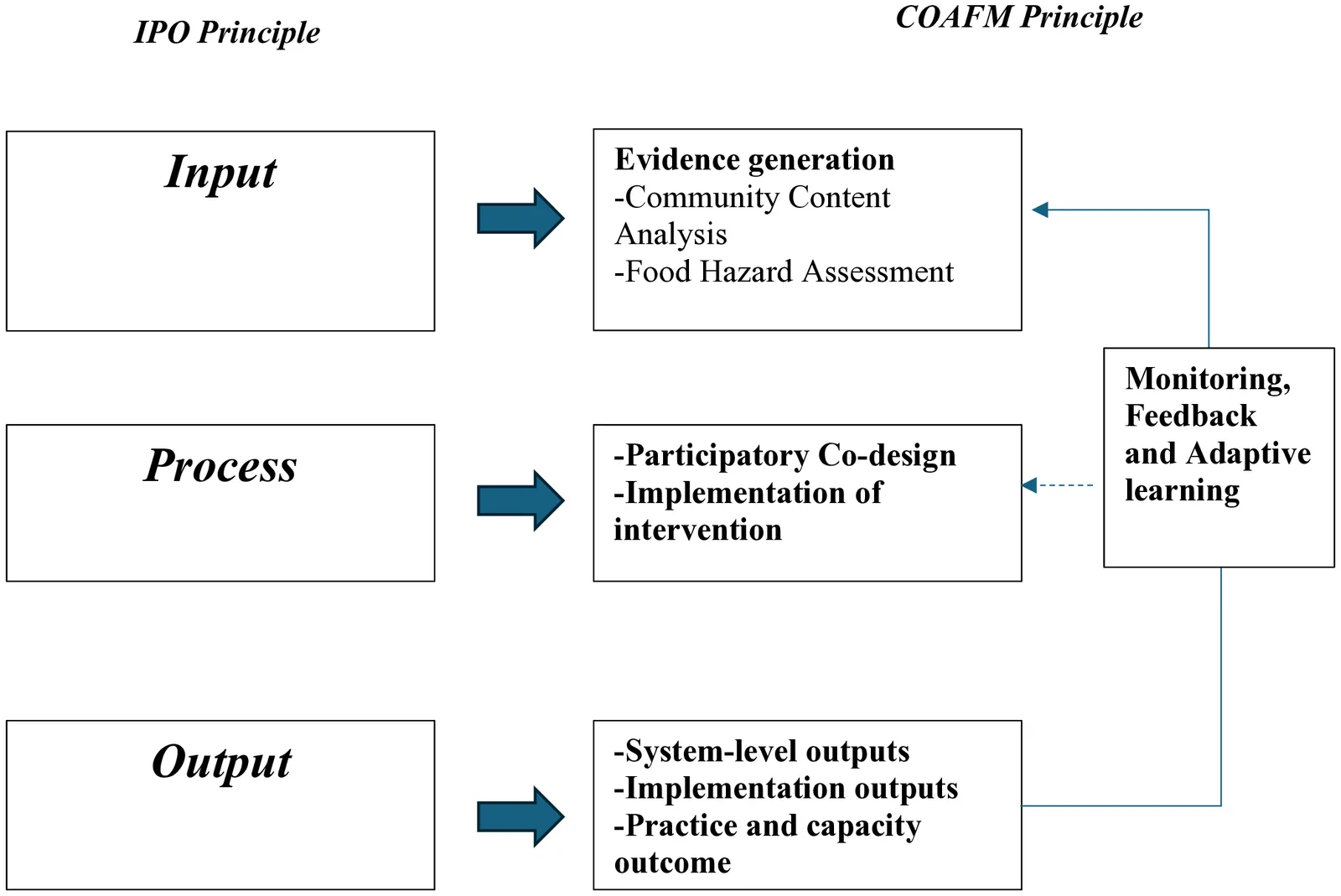
Conceptual framework of this study.

The conceptual framework integrates the COAFM with the Input–Process–Output (IPO) model (see Fig 1) [17,23–29,42]. The input component focuses on the generation of evidence through community content analysis and food hazard assessment, providing a context-specific understanding of food safety risks. The process component involves participatory co-design and implementation of intervention, emphasizing stakeholder engagement and locally adapted strategies. These processes led to outputs across three domains: system-level outputs, implementation outputs, and practice and capacity outcomes. In addition, feedback and learning were incorporated to support continuous adaptation and improvement of the food safety system within real-world community settings.

### Phase 1: Community content analysis

To conduct the community content analysis, both qualitative and quantitative data were collected using in-depth interviews and a structured household survey. In-depth interviews and a structured survey questionnaire were conducted for the community context analysis. Firstly, participants were purposively selected for the in-depth interviews based on a stakeholder analysis [43] which represented key stakeholders in the community food system, including: 1) farmers and local food producers (n = 3), 2) food vendors and restaurant operators (n = 3), 3) community leaders and village committee members (n = 3), 4) village health volunteers (n = 3), 5) public health officers and local authorities (n = 3), and 6) school representatives (n = 3). The interview topics included: 1) food supply and demand within the community, 2) knowledge related to food production, 3) support and subsidies for food safety management, and 4) individual and institutional support for food safety practices. The in-depth interviews were audio-recorded with participants’ permission and transcribed verbatim. The interview transcripts were reviewed several times to ensure accuracy and familiarity with the data. Qualitative data were analyzed manually using an inductive thematic analysis approach. Meaningful statements were identified, coded, and grouped into categories based on similarities in content. Related categories were then organized into broader themes that reflected participants’ experiences and perspectives regarding the implementation of the COAFM. The identified themes were reviewed and refined through discussion with the research team to ensure consistency and credibility [44,45].

**Fig 2:**
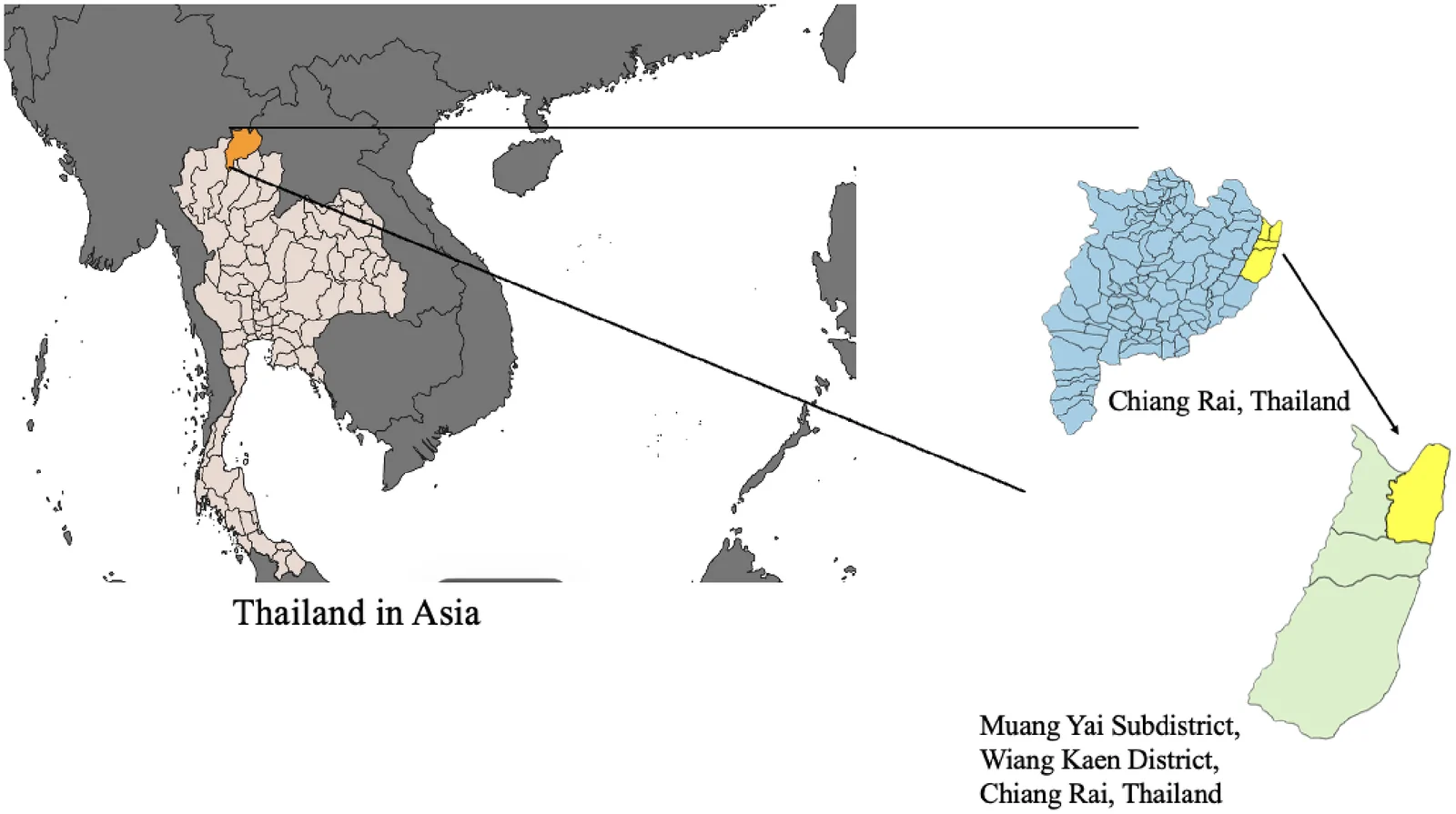
Study area showing the location of Muang Yai Subdistrict, Wiang Kaen District, Chiang Rai Province, Thailand. The figure illustrates the geographic location of the study area at three spatial scales: Thailand within Asia, Chiang Rai Province within Thailand, and Muang Yai Subdistrict within Wiang Kaen District, Chiang Rai Province. The boundary shapefiles obtained from the **Global Administrative Areas (GADM)** database (https://gadm.org).

In addition, the household survey was conducted in nine selected villages in Muang Yai Subdistrict, Wiang Kaen District, Chiang Rai Province, Thailand. Muang Yai Subdistrict (see Fig 2) was purposively selected as the pilot implementation site for the COAFM because it is a predominantly rural agricultural community where household food production and community-based food safety activities are common. The selected communities were considered appropriate for evaluating the early implementation of the COAFM model. The sampling frame consisted of all households located within the selected villages. The minimum required sample size was estimated a priori using G*Power version 3.1 for binary logistic regression (Z tests), assuming a two-tailed test, an anticipated odds ratio (OR) of 2.5 based on our previous epidemiological study, a significance level (α) of 0.05, a statistical power of 95%, and a baseline outcome probability of 0.20 [46]. The estimated minimum sample size was 109 households. Although the required minimum sample size was 109 households, all eligible households within the selected villages were approached for participation. Consequently, a total of 204 households were enrolled in the study, thereby exceeding the minimum sample size required for statistical analysis. Households were eligible for inclusion if they were located within the selected villages and had at least one adult household member (≥18 years) who was primarily responsible for food preparation or food production. One respondent was recruited from each household, with priority given to the primary food preparer or homemaker because this individual was considered the household member most familiar with household food preparation and food safety practices. Households were excluded if no eligible respondent was available at the time of the visit, if the household was unoccupied after repeated visits, or if the eligible respondent declined to participate. Village Health Volunteers (VHVs) accompanied the research team to approach all eligible households within the selected villages. Participation was voluntary, written informed consent was obtained from all respondents prior to data collection, and no eligible households declined participation.

**Fig 3:**
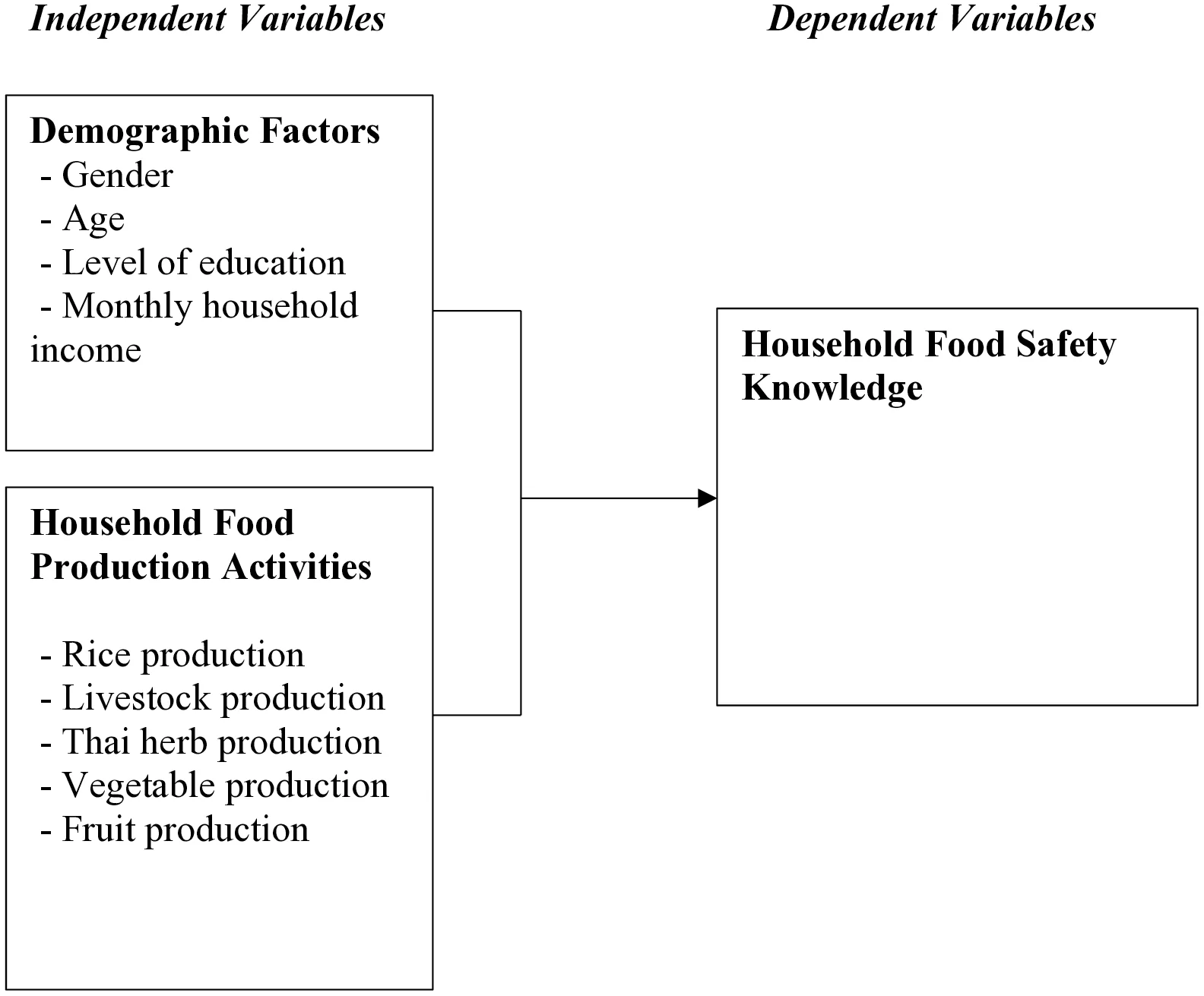
Conceptual framework of household survey variables.

The household questionnaire structures (see Fig 3 and S1 Table) consisted of two main independent variables including; 1) demographic factors including the gender, age, level of education, and average monthly income; 2) household food production activities included rice production, livestock production, Thai herb production, vegetable production, and fruit production. The dependent variable was household food safety knowledge scored as 1 for a correct response and 0 for an incorrect response. The reliability of the questionnaire was evaluated using the Kuder–Richardson Formula 20 (KR-20), yielding a coefficient of 0.75 [47]. The total score was calculated by summing the scores of all items. Participants were subsequently classified into two groups based on the median score, with scores below the median categorized as low household food safety and scores equal to or above the median categorized as high household food safety [46]. Descriptive statistics, including frequencies, percentages, means, and standard deviations, were used to summarize participant characteristics. Bivariate analyses using Chi-square tests were initially performed to examine the association between each independent variable and household food safety knowledge. Variables with p-values < 0.25 in the bivariate analysis were entered into an initial multivariable binary logistic regression model following the purposeful selection approach described by Bursac et al. [48]. According to the purposeful selection approach described by Bursac et al.[48], variables with p-values > 0.10 that did not act as confounders were removed, and the remaining variables were retained in the final multivariable logistic regression model. Adjusted odds ratios (AORs) with 95% confidence intervals (CIs) were reported, and statistical significance was determined at p < 0.05. Model fit was assessed using the Hosmer–Lemeshow goodness-of-fit test, and model explanatory power was evaluated using the Nagelkerke pseudo-R² statistic [46]. All statistical analyses were performed using IBM SPSS Statistics version 30 (IBM Corp., Armonk, NY, USA).

Finally, findings from the qualitative and quantitative components were integrated to provide a comprehensive community context assessment and to inform the development of the COAFM intervention.

### Phase 2: Food hazard assessment

All food establishments in the community (n = 8) were included in this assessment. The food hazard assessment was conducted in accordance with the Thailand SAN PLUS standard [49], focusing on both chemical and microbiological contamination. Chemical hazards included borax, formalin, salicylic acid, sodium hydrosulfite, and pesticide residues. Microbiological hazards focused primarily on *E. coli* contamination on food handlers’ hands, drinking water, and food utensils [50].

For chemical contamination, screening test kits were used as follows: 1) GPO™ borax test kits for detecting borax in ground pork, meatballs, and poultry; 2) GPO™ formalin test kits for detecting formalin in animal entrails; 3) GPO™ salicylic acid test kits for curry paste and garlic; 4) GPO™ sodium hydrosulfite test kits for bean sprouts and pickled bamboo shoots; and 5) MJPK test kits for pesticide residues in vegetables and fruits [51].

In the microbiological analysis, *E. coli* contamination in hand swabs, drinking water, and food utensils were assessed using the Most Probable Number (MPN) method, following the Bacteriological Analytical Manual (BAM) [52]. Presumptive positive samples were further confirmed using Eosin Methylene Blue (EMB) agar [35]. All samples were analyzed in triplicate (n = 3) to ensure analytical reliability.

### Phase 3: Participatory co-design and implementation

Participatory co-design was conducted to develop context-specific food safety strategies collaboratively based on findings from the previous phases. Key stakeholders were purposively invited based on the stakeholder analysis to represent the community food system, including: farmers and local food producers (n = 3), food vendors and restaurant operators (n = 3), community leaders and village committee members (n = 3), village health volunteers (n = 3), public health officers and local authorities (n = 3), and school representatives (n = 3). In addition, approximately 50 community members were invited to participate in broader consultation activities to ensure inclusiveness and community representation.

The co-design process was conducted through focus group discussions (FGDs), during which participants collaboratively identified priority food safety issues, discussed feasible solutions, and co-developed locally appropriate interventions [53,54]. The discussions were facilitated by the research team and structured around key themes derived from the community context analysis and food hazard assessment.

Following the co-design phase, the agreed strategies were implemented within the community. These included the establishment of local food safety initiatives, training activities for different stakeholder groups, and the development of community-based practices to improve food safety [55]. The implementation process emphasized stakeholder engagement, shared responsibility, and alignment with existing community structures [56].

### Phase 4: Monitoring, feedback and adaptive learning

Monitoring and feedback were conducted to assess the implementation of the COAFM. Follow-up activities were carried out through small group meetings with members of the community food safety committee. These meetings aimed to review the implementation progress, identify challenges and barriers encountered during the activities, and facilitate collective reflection among stakeholders. Participants collaboratively discussed lessons learned and proposed recommendations for improving future implementation. This process supported adaptive learning and continuous refinement of the food safety system within the community context [57–59].

### Ethical consideration

This study received ethical approval from the Ethics Committee in Human Research of the Chiang Rai Public Health Provincial Office (experimental protocol no. CRPPH0 NO.31/2563). All participants provided written informed consent prior to participation. The study objectives and procedures were explained to participants, who then signed the informed consent form before data collection. The inclusion criterion was individuals who had resided in the study area for at least one year. The exclusion criteria were participants who did not complete the questionnaire in full or were unable to provide information relevant to the study. In addition, numerical codes were used to anonymize the participants’ information. The participants were recruited between 15/02/2021 and 25/12/2021.

## Results

### Input: Evidence generation

The qualitative findings from in-depth interviews with relevant stakeholders and key informants involved in food safety management revealed that residents in the community have the capacity to grow vegetables for household consumption, including home-grown and indigenous vegetables. However, the primary economic crop in the area is pomelo, which typically requires chemical inputs to meet market demands. While many households cultivate vegetables for their own consumption, certain food products are regularly purchased from local markets, particularly the fresh market located in Wiang Kaen. Some farmers continue to engage in commercial or large-scale agriculture to generate income for their families. At the same time, knowledge of crop rotation practices remains limited among farmers. Nevertheless, some farmers have begun transitioning to organic farming, motivated by the desire to improve household health. In terms of support and institutional arrangements, financial and organizational mechanisms for food safety were found to be available at the local level. These include funding from local government authorities and the National Health Security Office (NHSO), as well as technical support from community hospital staff and local administrative officers. However, most stakeholders were unaware of these mechanisms, which limited their effective utilization and hindered coordinated food safety management within the community.

**Fig 4:**
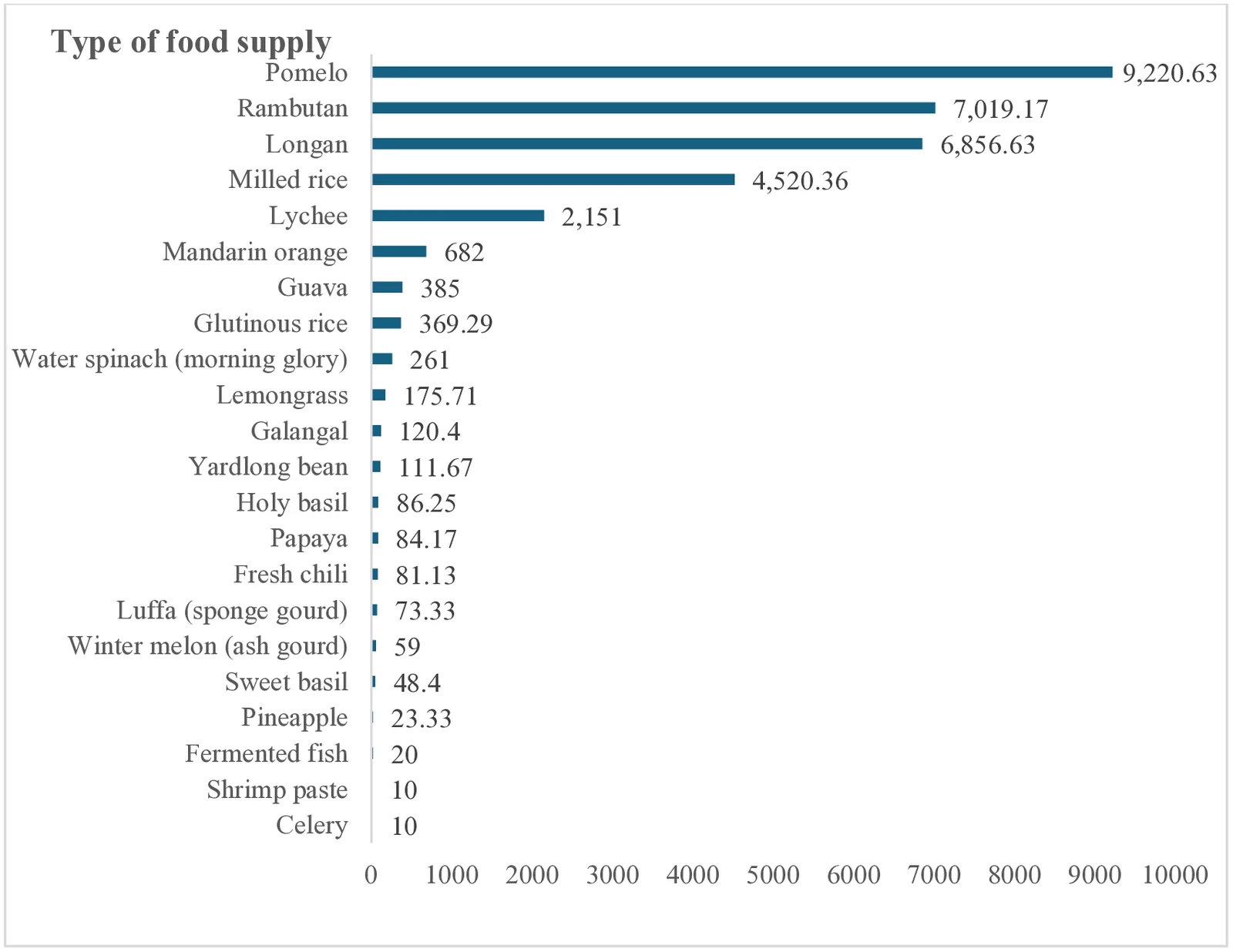
Average annual household food production in the community study (Unit: kg/household/year).

**Fig 5:**
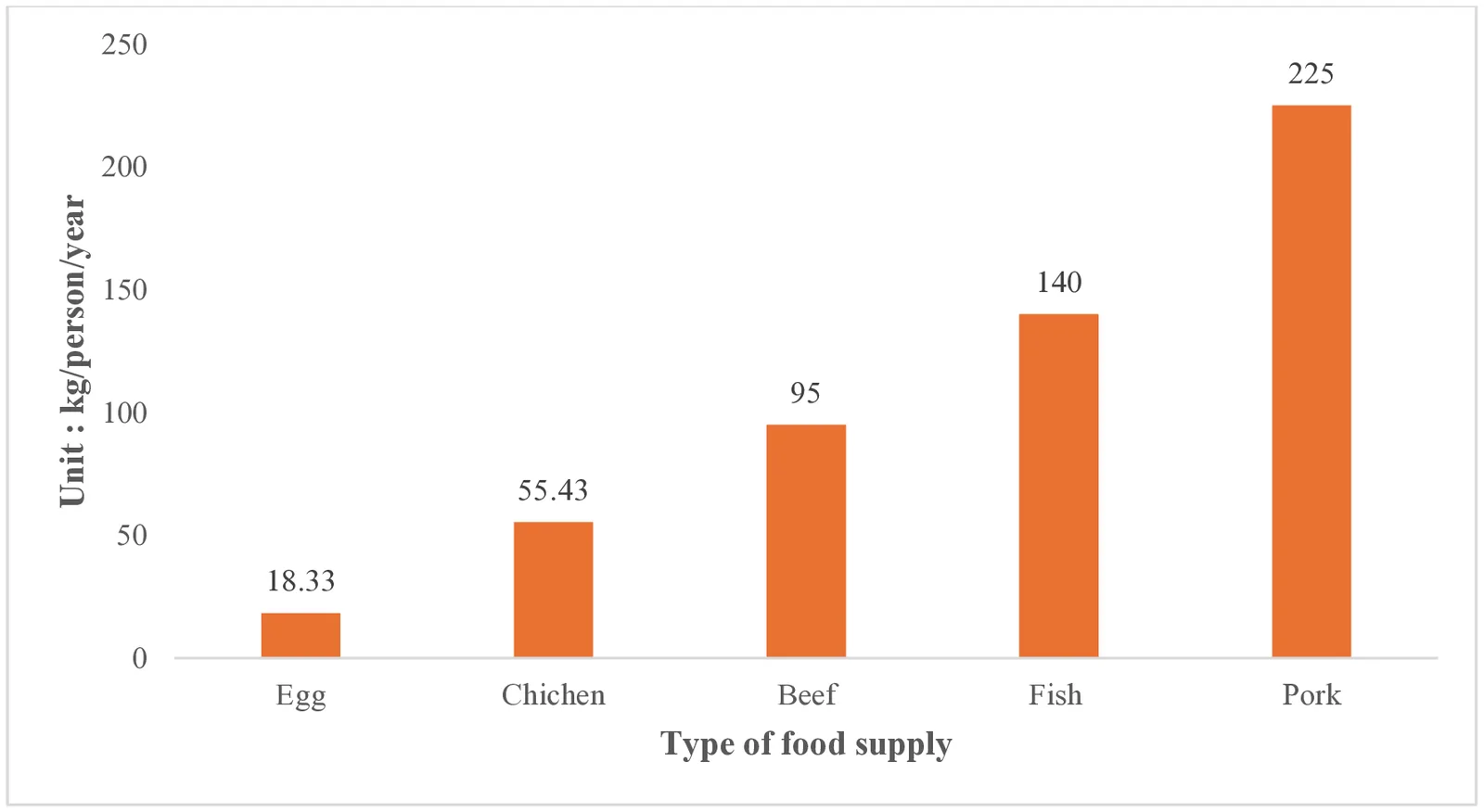
Average annual household food consumption in the community study (Unit: kg/person/year).

Food production assessment (Fig 4) revealed that rice was the most commonly cultivated crop, with 149 households (72.7%) reporting rice production, followed by pomelo (115 households; 56.1%) and lemongrass (66 households; 32.2%). In terms of production volume, pomelo had the highest yield (9,220.63 kg/household/year), followed by rambutan (7,019.17 kg/household/year) and longan (6,856 kg/household/year). In contrast, celery and shrimp paste had the lowest production volumes (10 kg/household/year), followed by fermented fish (20 kg/household/year). In addition, several food products were identified as foods with high consumption levels (Fig 5), particularly animal-based products such as pork, beef, and chicken, which are not produced locally. The average annual consumption per person per year was estimated at 225 kg for pork, 95.5 kg for beef, and 55.43 kg for chicken. This mismatch suggests a reliance on externally sourced food products, which may increase vulnerability to food safety risks that are beyond community-level monitoring and control.

Chemical and microbiological assessments conducted in eight food establishments showed that only one food shop had pesticide residues detected by the MJPK test-kits in garlic samples, which were sourced externally rather than produced within the community. Moreover, no microbiological contamination, including *E. coli*, was detected in samples collected from food handlers’ hands, drinking water, or food utensils. Overall, chemical and microbiological assessments indicated generally low contamination levels, although pesticide residues detected in externally sourced garlic highlighted potential risks associated with food supply chains beyond the local production system.

A total of 204 households participated in the survey. Most respondents were 30 years’ old or younger (46.6%), had a monthly household income of at least 5,000 THB (55.4%), and had completed primary school (61.3%). Regarding household food production, most households produced rice (76.5%) and fruit (67.2%), while 50.0% produced vegetables. Based on the household food safety knowledge assessment, 58.3% of the respondents were classified as having high household food safety knowledge (scores equal to or above the median). These findings are shown in S2 Table.

Bivariate analyses using Chi-square tests identified five candidate variables (gender, age group, rice production, vegetable production, and livestock production) with p-values < 0.25, which were subsequently entered into the initial multivariable binary logistic regression model. Following the purposeful selection approach, three variables remained in the final model: gender, age group, and rice production (see Table 1). The final model (see Table 2) demonstrated a good fit to the data (Hosmer–Lemeshow test, *p* = 0.708), explained 27.4% of the variance in household food safety knowledge (Nagelkerke R² = 0.274), and correctly classified 69.6% of the households. In the final multivariable model, female respondents had significantly lower odds of having higher household food safety knowledge than the male respondents (AOR = 0.52, 95% CI: 0.28–0.98, *p* < 0.05). Increasing age was significantly associated with lower odds of having high household food safety knowledge (AOR = 0.52, 95% CI: 0.40–0.68, *p* < 0.001). In addition, households producing rice had significantly lower odds of having high household food safety knowledge than households that did not produce rice (AOR = 0.21, 95% CI: 0.09–0.52, *p* < 0.001). The multivariable analysis showed that gender, age, and rice production were significantly associated with household food safety knowledge. These findings suggest that household characteristics should be considered when implementing community-based food safety interventions, as different household groups may require tailored communication strategies and capacity-building activities.

**Table 1:** Variable Selection Process for Multivariable Binary Logistic Regression Analysis.

Factors Associated with Household Food Safety Knowledge	p-Value of Bivariate analysis (Chi-square)	Initial multivariable model AOR (95% CI)	p-Value of Initial multivariable model AOR	Selection for final multivariable model AOR
**Demographic Variables**				
Gender	0.072	1.98 (1.21–3.80)	0.041	Yes
Age	<0.001	0.63 (0.48–0.84)	0.002	Yes
Level of education	0.697	-	-	No
Monthly household income	0.981	-	-	No
**Household Food Production Activities**				
Rice production	<0.001	0.18 (0.07-0.45)	<0.001	Yes
Vegetable production	0.065	0.93 (0.39-2.23)	0.866	No
Livestock production	0.160	0.49 (0.12-2.02)	0.325	No
Thai herb production	0.288	-	-	No
Fruit production	0.980	-	-	No

**Note:** Variables with p-values < 0.25 in the bivariate (Chi-square) analysis were entered into the initial multivariable binary logistic regression model following the purposeful selection approach. Variables with p-values < 0.10 in the initial multivariable model were retained in the final model.

**Table 2:** Final Multivariable Binary Logistic Regression Model for Factors Associated with Household Food Safety Knowledge.

**Factors Associated with Household Food Safety Knowledge**	**Model Analysis by Binary Logistic Regression**		
	**AOR**	***p*-Value**	**95% CI**
**Independent Variables**			
Gender	1.93	0.043	1.02-3.63
Age	0.52	<0.001	0.40-0.68
Rice production	0.21	<0.001	0.09-0.52
**Model Performance**			
Nagelkerke R^2^	0.274		
Classification Accuracy (%)	69.6		
Hosmer–Lemeshow test (p-Value)	0.708		

**Abbreviations:** AOR = Adjusted Odds Ratio; CI = Confidence Interval

### Process: Participatory co-design and intervention implementation

Through participatory meetings, stakeholders collaboratively developed a COAFM tailored to the local context. The process began with community-led assessments of safe food availability and food contamination risks, with particular emphasis on chemical contamination, while microbiological testing was conducted in collaboration with local institutions, such as hospitals and academic organizations. The findings from these assessments were communicated back to the community to support collective decision-making. Based on this evidence, community members identified priority actions to reduce food safety risks and improve local food systems. A community food safety committee was subsequently established as a key governance mechanism to coordinate and drive implementation. Committee members were nominated through a participatory process to ensure community acceptance and representation, including community leaders, farmers and local food producers, food vendors and restaurant operators, community members, village health volunteers, public health officers, local authorities, and school representatives. The committee was responsible for planning and implementing food safety activities, supported by funding from local government authorities and the NHSO, thereby enabling coordinated implementation and local ownership. Continuous communication between the committee and the community was maintained to ensure that activities aligned with local needs, with adjustments made when necessary, particularly in accordance with requirements for NHSO funding support.

**Fig 6:**
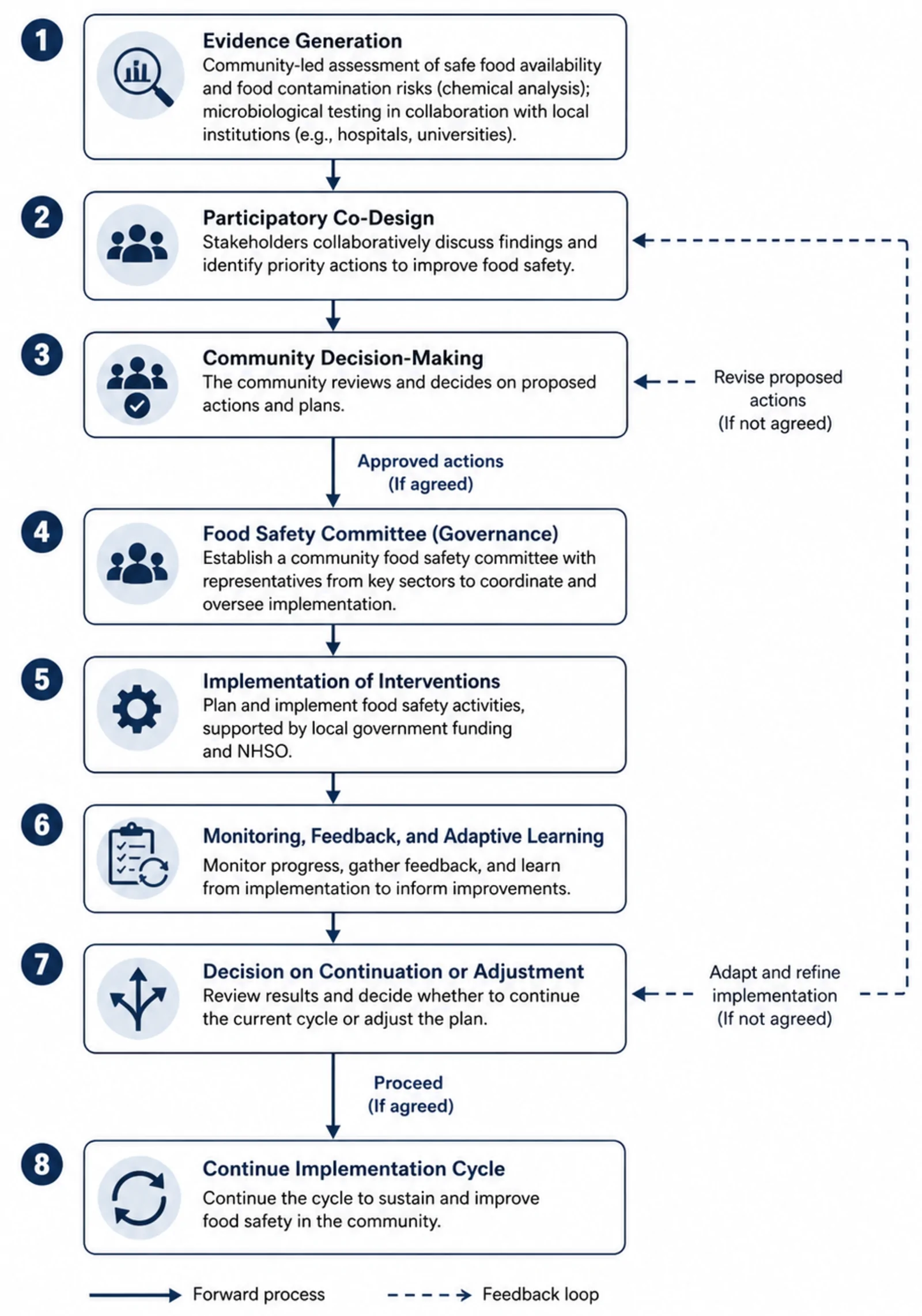
Process of the COAFM developed and implemented in the community study through participatory co-design^1^. ^1^ Artificial intelligence (AI) tools were used to assist in figure design, with final revisions made by the authors.

Fig 6 illustrates the COAFM process in this community following the participatory co-design phase.

### The early implementation outputs

**Fig 7:**
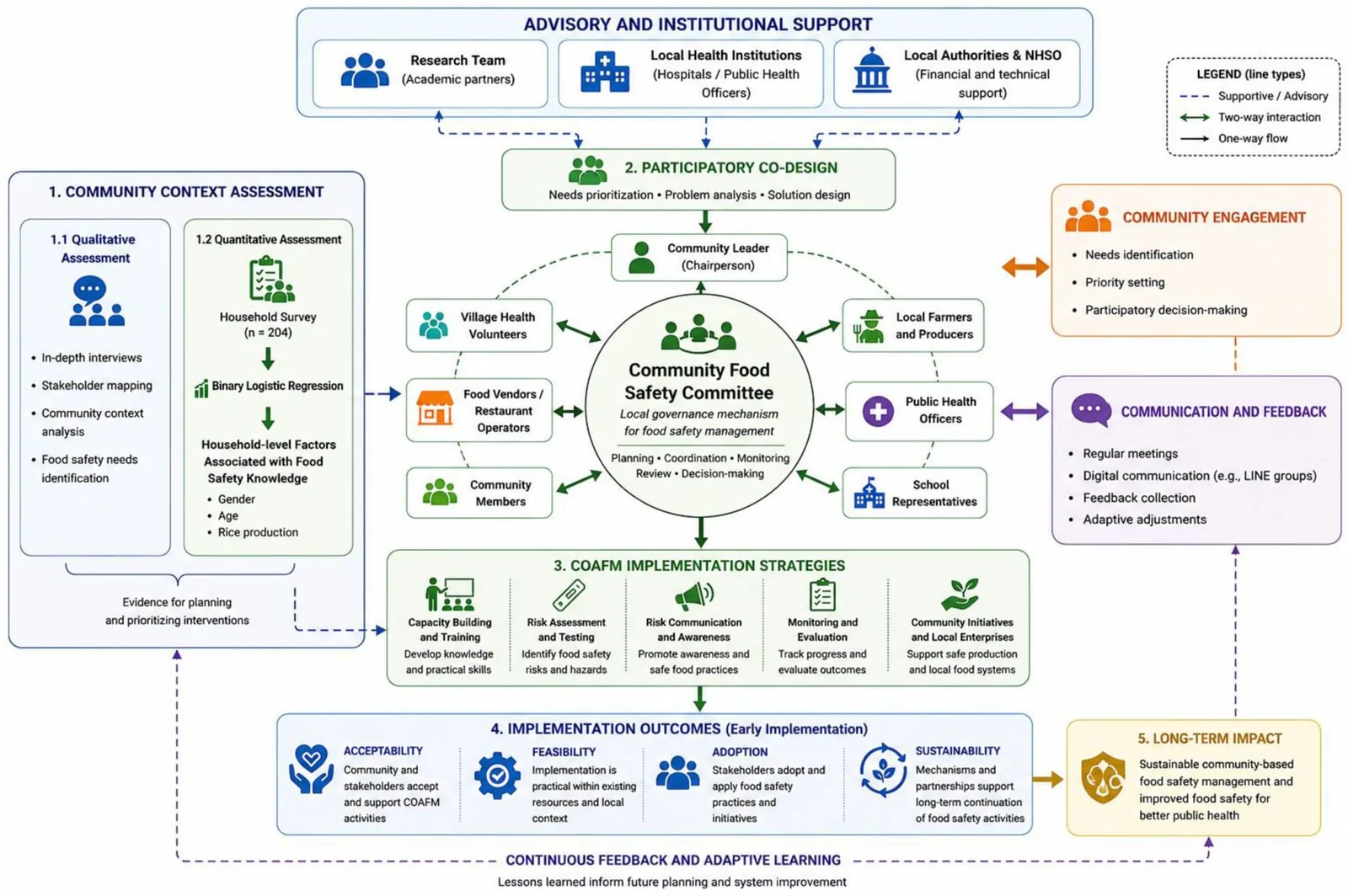
Governance Structure of the Community Food Safety Committee under the COAFM^1^. ^1^ The design of the figure was supported using AI-assisted visualization tools, followed by manual revision and validation by the authors.

Following the participatory co-design process, a Community Food Safety Committee (Fig 7) was established to coordinate, oversee, and monitor food safety activities within the community. The committee consisted of representatives from community leaders, village health volunteers, food vendors and restaurant operators, local farmers, community members, and schools, with technical support provided by the research team and local authorities. The establishment of this multi-stakeholder committee suggests that the COAFM was well accepted by key community stakeholders who facilitated collaborative governance for community food safety management.

The implementation of the COAFM was supported through existing community structures and local partnerships, indicating its feasibility within the study setting. Capacity-building activities, including food safety training and hands-on instruction in the use of rapid test kits for detecting chemical contaminants, were delivered to students and community members to strengthen food safety knowledge and practical skills. Regular meetings and digital communication platforms were also used to coordinate activities, monitor implementation progress, and provide continuous feedback among stakeholders.

Evidence of community adoption was reflected in several locally initiated activities following implementation, including the promotion of chemical-free vegetable production in schools, training programs for local farmers on safe food production, and the establishment of a community enterprise for safe agricultural products. These initiatives were developed and implemented through collaboration with community members and local organizations.

Overall, the findings suggest that the early implementation of the COAFM was acceptable and feasible within the community study. The integration of existing community organizations, multi-stakeholder participation, and continuous coordination mechanisms supported collaborative implementation and adaptive learning, while the establishment of the Community Food Safety Committee provided an organizational structure with the potential to support the long-term sustainability of community-based food safety management.

In addition, the household survey identified several household-level factors associated with food safety. Binary logistic regression analysis demonstrated that gender, age, and rice production were significantly associated with household food safety. These findings provide evidence that household characteristics should be considered when planning and implementing community-based food safety interventions, allowing educational activities and risk communication strategies to be tailored to different household groups.

## Discussion

This study developed and conducted the early implementation of a Community-Oriented Approach to Food Safety Management (COAFM) within a rural Thai community. The findings demonstrate that food safety management in rural settings extends beyond technical contamination control and is closely connected to local food systems, stakeholder coordination, governance structures, and community participation. Although contamination levels were generally low, the study identified important structural vulnerabilities, including dependence on externally sourced food products, limited awareness of institutional support mechanisms, and the dominance of economically driven agricultural practices that may rely on chemical inputs. These findings highlight the complexity of food safety management in community settings and support the need for context-specific and participatory approaches.

The multivariable binary logistic regression analysis identified several household characteristics associated with household food safety. Female respondents were more likely to have higher household food safety knowledge than male respondents, whereas increasing age and household rice production were associated with lower odds of having a high level of food safety knowledge. These findings suggest that household food safety is influenced not only by individual characteristics, but also by household livelihood patterns. Similar findings have been reported by fruit and vegetable producers in Ethiopia, where gender and education were associated with food safety and handling practices, highlighting the importance of considering demographic characteristics when designing food safety education program [60]. Likewise, previous studies have shown that food safety awareness varies across different age groups, indicating that educational interventions should be tailored according to the characteristics of target populations rather than adopting a uniform approach [61]. In addition, households engaged in rice production had lower odds of possessing high food safety knowledge, which may reflect differences in agricultural practices, traditional production systems, and access to food safety information. Previous studies have suggested that farming characteristics and socioeconomic conditions influence food safety-related practices, supporting the need to consider livelihood characteristics when planning community-based food safety interventions [62]. Within the COAFM framework, these findings provide locally generated evidence to support targeted educational activities and risk communication strategies for specific household groups, thereby strengthening evidence-informed and context-specific implementation.

The findings related to food demand and supply suggest that rural food systems may remain vulnerable despite strong local food production capacity. While households were able to produce vegetables for household consumption, highly consumed animal-based products were largely sourced from external markets. Similar to the study of Wynn et al. [63] and Komarek et al., 2021 [64], patterns of dependence on external food supply chains have been reported in other rural and low-resource settings. This mismatch may increase exposure to food safety risks beyond the control of local monitoring systems and reflects broader challenges associated with decentralized and informal food supply chains. Similar concerns have been reported in studies from other low- and middle-income settings, where food safety risks are shaped not only by household practices, but also by market dependency, supply chain fragmentation, and limited institutional integration [65].

An important contribution of this study is the demonstration of a participatory and locally embedded governance mechanism for food safety management. Unlike conventional top-down food safety approaches that primarily emphasize regulatory compliance and inspection, the COAFM emphasized stakeholder participation, collaborative decision-making, and adaptive implementation within the local context according to the World Health Organization [22] and Sheth et al.[22]. The establishment of the Community Food Safety Committee enabled coordination among community members, local authorities, public health officers, schools, and food vendors, thereby supporting shared responsibility and local ownership of food safety activities. This is consistent with the study of Chakma et al. [6], which found that awareness of food contamination risks may encourage community members to engage more actively in identifying locally appropriate solutions to protect health and strengthen food safety practices.

According to the study of Mansuri & Rao [21], the early implementation process also highlighted the importance of adaptive learning and continuous stakeholder engagement in sustaining community-oriented food safety activities. Through regular communication, feedback meetings, and collaborative problem-solving, implementation strategies could be adjusted according to local needs and resource availability. This iterative process reflects principles commonly emphasized in implementation science and community-oriented public health approaches, where sustainability depends on local participation, institutional support, and the capacity to adapt interventions to dynamic community conditions. Importantly, within the COAFM framework, these findings were not used solely to identify associated factors, but also to support evidence-informed intervention planning. The household survey and multivariable analysis provided locally generated evidence that enabled the Community Food Safety Committee to identify priority population groups and tailor capacity-building activities according to the characteristics of the community. This evidence-informed approach strengthens participatory decision-making and adaptive implementation, which are fundamental components of the COAFM. By integrating quantitative evidence with stakeholder engagement and qualitative community context analysis, the COAFM facilitated the development of locally appropriate food safety interventions that were more responsive to community needs and therefore more likely to achieve long-term sustainability.

However, several limitations should be considered when interpreting the findings of this study. First, the study focused primarily on framework development and early implementation rather than a formal evaluation of its effectiveness; therefore, long-term impacts on food safety behaviors, contamination reduction, and health outcomes could not be determined. Second, the study was conducted within a specific rural community context in northern Thailand, which may limit generalizability to other settings. Third, contamination levels observed during the assessment period were relatively low, limiting the ability to assess broader environmental and health impacts associated with food safety risks.

Despite these limitations, the study provides an initial implementation framework for strengthening food safety management in rural community settings. The findings suggest that integrating evidence generation, participatory governance, and adaptive implementation processes may support more sustainable and context-appropriate food safety systems in resource-limited environments. Future studies should evaluate long-term implementation outcomes, behavioral changes, and health impacts associated with community-oriented food safety management approaches.

## Conclusion

This study developed and conducted the early implementation of a Community-Oriented Approach to Food Safety Management (COAFM) within a rural Thai community. The findings demonstrate that food safety management in community settings involves not only contamination control, but also broader issues related to local food systems, stakeholder coordination, governance structures, and adaptive implementation processes. The COAFM provided a participatory and locally embedded framework that supported collaborative decision-making, community engagement, and coordination among multiple stakeholders involved in food safety management.

Although contamination levels observed during the study were generally low, the findings highlighted important structural vulnerabilities associated with dependence on externally sourced food products, limited institutional awareness, and fragmented coordination mechanisms. The study suggests that integrating evidence generation, participatory governance, and adaptive learning processes may support more sustainable and context-appropriate food safety management in resource-limited rural settings.

Overall, the COAFM demonstrates the potential of community-oriented and participatory approaches to strengthen local food safety systems beyond conventional top-down regulatory models. Future studies should further evaluate long-term implementation outcomes, behavioral changes, and health impacts associated with this approach in different community contexts.

## Supporting information

S1 Table The household questionnaire structures. (PDF)

S2 Table The percentage result of the demographic factors. (PDF)
