# Supplementary material for: Development and implementation of a community-oriented approach to food safety management in a Rural Thai community
Source: PLoS One. 2026 Aug 27;21(8):e0350910. doi: 10.1371/journal.pone.0350910 (PMC13521373; doi:10.1371/journal.pone.0350910)
Supplement: Supplementary file 1 [file pone.0350910.s001.pdf]

S1 Table. The household questionnaire structures

Section 1. Demographic Characteristics

Instructions: Please answer the following questions by selecting the most appropriate response.

1. Gender

☐ Male

☐ Female

2. Age

☐ Less than 30 years

☐ 31–40 years

☐ 41–50 years

☐ 51–60 years

☐ More than 60 years

3. Highest Level of Education

☐ No formal education

☐ Primary school

☐ Secondary school

☐ High school / Vocational certificate

☐ Bachelor's degree or higher

4. Average Monthly Household Income

☐ Less than 5,000 THB

☐ 5,001–10,000 THB

☐ 10,001–15,000 THB

☐ 15,001–20,000 THB

☐ More than 20,000 THB

Section 3. Household Food Production Activities

Instructions: Please indicate whether your household is involved in the following activities.

5. Rice Production

☐ Yes , The amount of this production……………………………….kg/household/year

The specific type of this production is…………………………………………………

………………………………………………………………………………………………

☐ No

6. Livestock Production

(raising cattle, buffaloes, pigs, chickens, ducks, or other livestock)

☐ Yes , The amount of this production……………………………….kg/household/year

The specific type of this production is…………………………………………………

………………………………………………………………………………………………

☐ No

7. Thai Herb Production

(cultivation of medicinal or culinary herbs)

☐ Yes , The amount of this production……………………………….kg/household/year

The specific type of this production is…………………………………………………

………………………………………………………………………………………………

☐ No

8. Vegetable Production

☐ Yes , The amount of this production……………………………….kg/household/year

The specific type of this production is…………………………………………………

………………………………………………………………………………………………

☐ No

9. Fruit Production

☐ Yes , The amount of this production……………………………….kg/household/year

The specific type of this production is…………………………………………………

………………………………………………………………………………………………

☐ No

Section 3. Household Food Consumption

Instructions: Please indicate whether your household is consumed in the following activities.

10. Rice Comsumption

☐ Yes , The amount of this consumption……………………………….kg/household/year

The specific type of this production is…………………………………………………

…………………………………………………………………………………………

☐ No

11. Livestock comsumption

(raising cattle, buffaloes, pigs, chickens, ducks, or other livestock)

☐ Yes , The amount of this consumption……………………………….kg/household/year

The specific type of this production is…………………………………………………

…………………………………………………………………………………………

☐ No

12. Thai Herb Consumption

(cultivation of medicinal or culinary herbs)

☐ Yes , The amount of this consumption……………………………….kg/household/year

The specific type of this production is…………………………………………………

…………………………………………………………………………………………

☐ No

13. Vegetable Consumption

☐ Yes , The amount of this consumption……………………………….kg/household/year

The specific type of this production is…………………………………………………

…………………………………………………………………………………………

☐ No

14. Fruit Consumption

☐ Yes , The amount of this consumption……………………………….kg/household/year

The specific type of this production is…………………………………………………

…………………………………………………………………………………………

☐ No

Section 4. Household Food Safety Knowledge

Instructions: Please indicate whether each of the following statements is **True** or **False**.

4.1 Eating contaminated or unhygienic food can cause diarrhea.
☐ True ☐ False

4.2 It is acceptable to wear rings while preparing food.
☐ True ☐ False

4.3 Cooked food should be left at room temperature for an extended period before consumption.
☐ True ☐ False

4.4 Borax is commonly found in minced pork.
☐ True ☐ False

4.5 Formalin is sometimes found in certain seafood products, such as squid and shellfish.
☐ True ☐ False

4.6 Mold inhibitors or preservatives are commonly found in dried food products, curry paste, and garlic.
☐ True ☐ False

4.7 White-colored bean sprouts are always safe for consumption.
☐ True ☐ False

4.8 Cooking oil can be safely reused for deep-frying more than three times.
☐ True ☐ False

4.9 Baking soda cannot be used to help reduce pesticide residues on fruits and vegetables.
☐ True ☐ False

4.10 Hands should be washed thoroughly before every meal.
☐ True ☐ False
