# Supplementary material for: Development and implementation of a community-oriented approach to food safety management in a Rural Thai community
Source: PLoS One. 2026 Aug 27;21(8):e0350910. doi: 10.1371/journal.pone.0350910 (PMC13521373; doi:10.1371/journal.pone.0350910)
Supplement: Supplementary file 2 [file pone.0350910.s002.pdf]

S2 Table. The percentage result of the demographic factors

Frequency Table

Gender

		Frequency	Percent	Valid Percent	Cumulative Percent
Valid	Male	104	51.0	51.0	51.0
	Female	100	49.0	49.0	100.0
	Total	204	100.0	100.0	

Group of age

		Frequency	Percent	Valid Percent	Cumulative Percent
Valid	not over 30 years	95	46.6	46.6	46.6
	41- 50 years	71	34.8	34.8	81.4
	51-60 years	38	18.6	18.6	100.0
	Total	204	100.0	100.0	

Group of Income

		Frequency	Percent	Valid Percent	Cumulative Percent
Valid	Less than 5,000 THB per month	91	44.6	44.6	44.6
	5,000 THB per month to heighes	113	55.4	55.4	100.0
	Total	204	100.0	100.0	

Level of Graduation

		Frequency	Percent	Valid Percent	Cumulative Percent
Valid	No formal education	30	14.7	14.7	14.7
	Primary School	125	61.3	61.3	76.0
	Lower secondary school	25	12.3	12.3	88.2
	Higher secondary school	18	8.8	8.8	97.1

		Frequency	Percent	Valid Percent	Cumulative Percent
	Bachelor's degree or higher	6	2.9	2.9	100.0
	Total	204	100.0	100.0	

Rice Production

		Frequency	Percent	Valid Percent	Cumulative Percent
Valid	No	48	23.5	23.5	23.5
	Yes	156	76.5	76.5	100.0
	Total	204	100.0	100.0	

Livestcok Production

		Frequency	Percent	Valid Percent	Cumulative Percent
Valid	No	174	85.3	85.3	85.3
	Yes	30	14.7	14.7	100.0
	Total	204	100.0	100.0	

Thai herb Production

		Frequency	Percent	Valid Percent	Cumulative Percent
Valid	No	194	95.1	95.1	95.1
	Yes	10	4.9	4.9	100.0
	Total	204	100.0	100.0	

Vegetable Production

		Frequency	Percent	Valid Percent	Cumulative Percent
Valid	No	102	50.0	50.0	50.0
	Yes	102	50.0	50.0	100.0
	Total	204	100.0	100.0	

Fruit Production

		Frequency	Percent	Valid Percent	Cumulative Percent
Valid	No	67	32.8	32.8	32.8
	Yes	137	67.2	67.2	100.0
	Total	204	100.0	100.0	

Group of median knowledge

		Frequency	Percent	Valid Percent	Cumulative Percent
Valid	Less than the median	85	41.7	41.7	41.7
	Median value to hightest	119	58.3	58.3	100.0
	Total	204	100.0	100.0	
